# The Potential Applications of Raman Spectroscopy in Kidney Diseases

**DOI:** 10.3390/jpm12101644

**Published:** 2022-10-03

**Authors:** Charlotte Delrue, Marijn M. Speeckaert

**Affiliations:** 1 Department of Nephrology, Ghent University Hospital, 9000 Ghent, Belgium; 2 Research Foundation-Flanders (FWO), 1000 Brussels, Belgium

**Keywords:** kidney disease, Raman spectroscopy, surface-enhanced Raman spectroscopy

## Abstract

Raman spectroscopy (RS) is a spectroscopic technique based on the inelastic interaction of incident electromagnetic radiation (from a laser beam) with a polarizable molecule, which, when scattered, carries information from molecular vibrational energy (the Raman effect). RS detects biochemical changes in biological samples at the molecular level, making it an effective analytical technique for disease diagnosis and prognosis. It outperforms conventional sample preservation techniques by requiring no chemical reagents, reducing analysis time even at low concentrations, and working in the presence of interfering agents or solvents. Because routinely utilized biomarkers for kidney disease have limitations, there is considerable interest in the potential use of RS. RS may identify and quantify urinary and blood biochemical components, with results comparable to reference methods in nephrology.

## 1. Introduction

Biospectroscopy enables the identification of key biochemical changes in tissue associated with a given pathological state, facilitating biomarker extraction and automated detection of key lesions. This technology has the ability to generate a unique spectral fingerprint that is representative of the chemical bonds by utilizing the interaction of light with the constituent molecules present within any given biosample. This allows the cellular activity specific to any given pathological state to be identified. Infrared spectroscopy and Raman spectroscopy (RS) are two important analytical techniques, which are low-cost and label-free, with minimal sample preparation required [1]. RS, unlike infrared absorption, is well suited for biological tissue measurements due to its low sensitivity to water [2]. In comparison to biopsy and biomarkers, optical methods have the potential to develop noninvasive or minimally invasive and objective approaches to pathological assessment [3]. Furthermore, technological advances in chemometric analysis over the last decade have enabled a high throughput of large datasets, with an increased investigation of its potential application in renal medicine [1]. In the present review article, we give an overview of the current applications of RS in kidney diseases and highlight the unmet needs for further research.

## 2. Raman Spectroscopy

**Figure 1:**
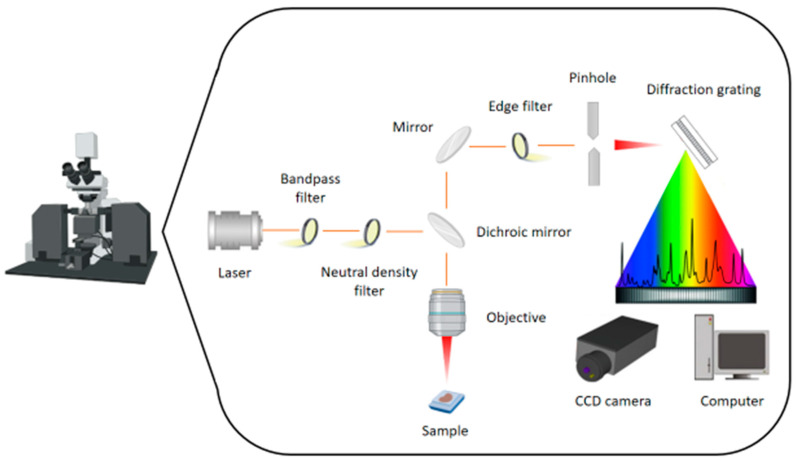
Schematic overview of a Raman microscopy/spectroscopy setup.

RS is based on the inelastic scattering of light by polarizable molecules, which reveals the vibrational energy levels of the molecular chemical bonds [4,5]. A schematic overview of an RS setup is presented in Figure 1.The magnitude of the change in molecular polarization is proportional to the intensity of Raman scattering. In classical physics, light is thought of as a wave with electric and magnetic fields. Each electromagnetic wave is made up of both an electric and a magnetic field that are perpendicular to one another. While RS under standard conditions provides information about a sample’s chemical composition, polarized RS can provide additional information, such as the symmetry of vibrational modes and the sample’s orientation [6]. Vertical variations in Raman intensities correspond to changes in component concentrations. Horizontal shifts in Raman signals are caused by minor differences in molecular vibrations caused by molecule composition changes [2]. 

**Figure 2:**
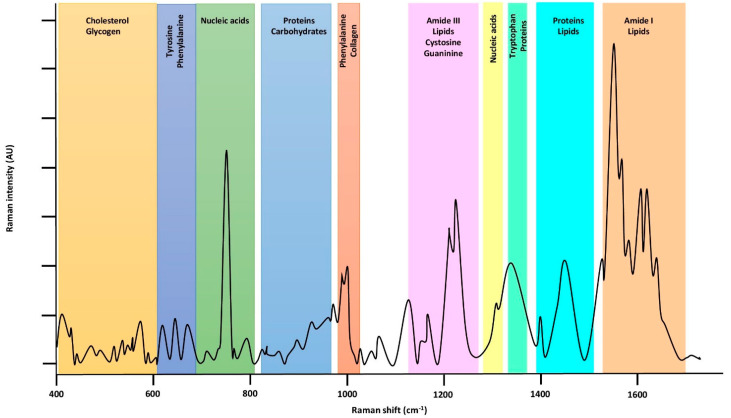
A typical biological Raman spectrum.

RS enables the analysis of chemical structures in biological fluids such as plasma, urine, and cerebrospinal fluid in a quick and nondestructive manner. Talari et al. published a large database of molecular fingerprints containing the most commonly observed peak frequencies and their assignments [7]. Figure 2 illustrates the Raman spectrum of whole blood over the range 400–1700 cm^−1^.

Previously, the application of RS was restricted due to its low sensitivity, high cost, and a lack of readily available, onsite analysis [6]. Recent RS technological advancements have resulted in a readily available, portable, and reasonably priced RS device [8]. Multivariate spectral analysis methods are frequently used to process Raman spectra and facilitate data interpretation, which could also be the case when diagnosing nephropathies [2].

Surface-enhanced Raman spectroscopy (SERS) is a subtype of RS that is specifically designed to increase its sensitivity of low concentration analytes (by factors of 10^4^ or higher, enabling single-molecule SERS). The addition of an enhancement material (e.g., colloidal metals and roughened metals, including gold, silver, and copper) to a sample significantly increases the electromagnetic fields of adsorbate molecules generated by the excitation of localized surface plasmons [9]. The combination of SERS with the tips of atomic force microscopy or scanning tunneling microscopy has resulted in tip-enhanced Raman scattering, a powerful imaging tool. The rich vibrational spectroscopic information provided by SERS distinguishes it from many other techniques for analytical applications [10]. SERS has numerous advantages over traditional methods. First of all, it is noninvasive. Second, SERS can distinguish multiple substances at the same time. Third, SERS may be a more convenient, less expensive, and faster tool than traditional techniques in clinical and biochemical laboratories [11]. On the other hand, the SERS technique has limitations in that (1) it requires intimate contact between the enhancing surface and the analyte, (2) the substrates degrade over time, resulting in a decrease in signal, (3) the substrates have limited selectivity for a given analyte, (4) the substrates have limited reusability, and (5) there are problems with homogeneity and reproducibility of the SERS signal within a substrate [12].

## 3. Raman Spectroscopy: A Novel Tool to Detect Renal Biomarkers?

### 3.1. Skin

Chronic kidney disease (CKD) is one of the most common noncommunicable disease pathologies. Kidney failure is associated with a variety of physiological and pathological characteristics of internal organs, all of which are closely related to the skin condition and affect its component composition [13]. One of the leading trends in therapeutic disciplines is the analysis of changes in the composition of different human skin layers. In skin analysis, RS is used to quantify the content of a specific component in the skin, determine dermal drug delivery, identify biophysical links between vibrational characteristics and specific compositional and chemical changes associated with aging, screen for skin cancer, etc. [14,15,16,17]. In a study of 85 hemodialysis patients (90 spectra) and 40 healthy adults (80 spectra) [18], investigation of the forearm skin using RS yielded an accuracy of 0.96, sensitivity of 0.94, and specificity of 0.99 in identifying the target subjects with kidney failure. Multivariate analysis of the Raman component of the skin spectrum proved to be specific for identifying spectral features associated with metabolic changes in the skin in kidney failure, with specificity, sensitivity, and accuracy of 0.91, 0.84, and 0.88, respectively, whereas the age factor had no significant effect on the analysis. When using the partial least square-discriminant analysis (PLS-DA) method to classify subjects on the basis of the presence of kidney failure, the most informative Raman spectral bands were 1315–1330 cm^−1^ (amide III, δ(CH2) in α-helix collagen) [19], 1450–1460 cm^−1^ (δ(CH) in proteins and lipids) [20], and 1700–1800 cm^−1^ (v(CO) in lipids and phospholipids, v(COO)) [21,22]. The analysis’s accuracy, sensitivity, and specificity are adequate for clinical use, making it a potential foundation for developing new methods of monitoring hemodialysis patients and screening the health status of patients with kidney failure [18]. Future research could concentrate on RS as a useful tool for objectively assessing treatment response in CKD patients. Pruritus, for example, is a major issue in both dialysis patients and those with advanced kidney failure. Investigating skin spectra in CKD patients with and without pruritus, as well as evaluating the change in Raman spectral bands after starting an anti-itch treatment, could be an intriguing and worthwhile area of research.

### 3.2. Urine

Research in the last decades has published several methods based on RS and SERS for the detection of urinary renal biomarkers, including urea peaks at 1004 cm^−1^ (symmetrical CN stretch) and 1161 cm^−1^ (attributed to NH_2_ modes), creatinine peaks at 608 cm^−1^ (NCH_3_ stretching, CO deformation and ring vibrations), 680 cm^−1^ (CNH_2_ and CO stretching, ring vibrations), 846 cm^−1^ (CNH_2_ deformation and ring vibrations), and 910 cm^−1^ (C–C–N stretching), glucose peak at 1128 cm^−1^ (C–O stretching), and protein peaks at 600, 850, 1060, and 1470 cm^−1^ (disulfide bonds, tyrosine, C–N bonds, and CH_2_ and CH_3_ angle bending) [23,24,25,26]. An overview of the most important findings of these studies is presented in Table 1 [11,27,28,29,30,31,32,33,34,35,36,37,38,39,40,41,42,43]. The applications of RS for the identification of uropathogens, urine crystals, and kidney stones are not included in this review.

A pilot study of renal allograft recipient Lewis rats and obese diabetic ZSF1 rats with kidney disease investigated if SERS could predict the number of biochemical substances in urine samples that are related to kidney function. PLS predicted the biochemical parameters of kidney function using the SERS spectra, resulting in R^2^ = 0.8246 (*p* < 0.001, urine protein), R^2^ = 0.8438 (*p* < 0.001, urine creatinine), R^2^ = 0.9265 (*p* < 0.001, urea), R^2^ = 0.8719 (*p* < 0.001, serum creatinine), and R^2^ = 0.6014 (*p* < 0.001, urine protein-to-creatinine ratio). SERS predicted urine creatinine within the biological range of 1–9 mmol/L with a root-mean-squared error of cross-validation (RMSEcv) of 0.69 mmol/L [44]. 

By analyzing urine from diabetic and hypertensive patients without and with complications compared to controls and correlating the changes in the spectral features, dispersive near-infrared RS proved to be a promising tool for analysis of these renal biomarkers. The intensity of specific peaks varied depending on the group, such as the absence of the glucose peak (1128 cm^−1^) for the healthy control group and its presence in all disease groups, and the decreased intensity of the urea (1004 cm^−1^) and creatinine (680 cm^−1^) peaks for the diseased groups compared to the control group. The first principal component analysis (PCA) loading vectors revealed urea, creatinine, and glucose spectral features. Urinary urea and creatinine concentrations decreased as the disease progressed from controls to lower/higher risk of complications and the dialysis group (PC1 score, p < 0.05). When urine of the lower/higher risk of complications group was compared to the control group, the amount of glucose increased (PC3 score, *p* < 0.05). The discriminating model had a higher overall classification rate of 70% (89% for the control group, and 81% for both the high risk for complications and the dialysis groups). The authors concluded that SERS could provide diagnostic information about kidney failure, as well as a better estimation of disease prognosis due to diabetes mellitus and hypertension using a single spectrum of urine [32]. 

A Chinese study with 126 CKD patients (CKD stages 2–5) and 97 healthy subjects compared the differences in SERS properties of urine [37]. Three Raman peaks at 640–680, 1006, and 1625–1655 cm^−1^ were also reported by others [39,45], while the missing peak at 1026/1028 cm^−1^ was probably too close to the strong peak at 1006 cm^−1^. Other prominent Raman signals at 1079, 1185, 1287, and 1383 cm^−1^ in the CKD group may be unique to this subset of the CKD population because they have not been reported previously [37]. Nitrogenous compounds (C–N stretching from primary amines) and cytosine may be represented by these peaks. Nitrogenous compounds are most likely produced by the urea cycle’s amino-acid metabolism and may be higher in the urine of CKD patients [46]. Cytosine is one of the four major bases in both DNA and RNA, and it is derived from cellular processes that are also linked to CKD progression [47]. For the urine spectra, CKD was distinguished from non-CKD controls with a sensitivity of 78.0%, specificity of 86.2%, and accuracy of 81.8% using PCA-LDA. The mean ± standard deviation (SD) for integration area under the receiver operating characteristic (ROC) curve for urine was 0.886 ± 0.025 (*p* < 0.0001). The various CKD stages were separated with a moderate accuracy of 75.4%. The distinction was not obvious, most likely because of the small number of samples in each group, which differed according to a variety of physiological or metabolic conditions. Furthermore, some of the Raman signals used in the discrimination analysis were not linearly related to the stages of CKD. The PLS prediction of urine spectra was 0.7335 for urine urea (*p* < 0.001), with peaks at 527, 1006, and 1159 cm^−1^, 0.7901 for urine creatinine (*p* < 0.001), with peaks at 605, 681, and 848 cm^−1^, 0.4644 for estimated glomerular filtration rate (eGFR) (*p* < 0.001), and 0.6579 for microalbuminuria (*p* < 0.001) [37]. 

A total of 235 urine specimens from healthy people were analyzed using Raman Chemometric Urinalysis (Rametrix^TM^) and showed significant differences (*p* < 0.001) in the Raman spectra of a urine specimen, which could be attributed to the age of the donor. Future research should focus on the influence of diet and lifestyle on these variations. Menstruation did not contribute statistically significant changes to the Rametrix^TM^ spectral signature of urine in the 30 day study subset of urine specimens [48]. Rametrix^TM^ [26] was used to compare Raman spectra from 362 urine specimens from patients receiving peritoneal dialysis (PD) therapy for end-stage kidney disease (ESKD), 395 dialysate specimens from those PD therapies, and 235 urine specimens from healthy human volunteers. The entire Raman spectrum of a specimen (i.e., chemometrics) could determine its type (i.e., urine or dialysate) with >98% accuracy, sensitivity, and specificity or the donor’s state (i.e., healthy human or PD patient) with better than 96% accuracy (with better than 97% sensitivity and 94% specificity). Rametrix^TM^ could potentially be used to determine whether PD’s disease therapies are patient-specific, whether there are differences between subsequent treatments, and how they affect the patient’s outcomes [49].

The detection and quantification of macro- and microhematuria in human urine samples have also been tested using RS. Anticoagulated whole blood was mixed with freshly collected urine to achieve blood/urine (*v*/*v*) concentrations of 0%, 0.25%, 0.5%, 1%, 2%, 6%, 10%, and 20%. Raman spectra were obtained at 785 nm, and data were analyzed using Rametrix^TM^. With prediction accuracies of 91% and 94%, discriminate analysis of principal component (DAPC) models was capable of detecting various levels of microhematuria in unknown urine samples. These preliminary findings indicate that RS and chemometric analyses can be used to detect and quantify micro- and macrohematuria in clinically relevant urine specimens that have not been processed [43].

### 3.3. Serum

Serum creatinine and urea are important biomarkers for diagnosing and monitoring kidney disease. Several studies have investigated the use of RS and SERS to detect these serum biomarkers rapidly and sensitively (Table 2) [11,28,37,50,51,52,53,54,55,56]. 

In an exploratory study, there were several obvious differences between normal (n = 50) and CKD serum samples (n = 60) in the integration areas of Raman spectral wavebands within 550–1750 cm^−1^. Although there were significant SERS spectral differences between the two groups, primary SERS peaks were observed in both groups at 641, 724, 813, 1003, 1132, 1210, 1326, 1450, 1583, and 1655 cm^−1^, with the strongest signals at 641, 724, 1326, 1450, and 1655 cm^−1^. SERS intensity peaked at 724, 1326, and 1450 cm^−1^ in the normal group but not in the patients. The intensity at 641 and 1655 cm^−1^ was higher in CKD patients than in the control group. Nucleic acids (641, 724, 813, 1003, 1210, 1132, and 1450 cm^−1^), carbohydrates (641, 890, and 1094 cm^−1^), and lipids (1278 and 1327 cm^−1^) were identified as the major SERS peaks. The CKD group had a higher peak at 1655 cm^−1^ (amide I band) than the normal group, indicating more proteins in the α-helix conformation. The SERS peak at 724 cm^−1^ caused by hypoxanthine showed a low signal in CKD, indicating an abnormal metabolism of DNA or RNA bases. The decreased intensity of the 1326 cm^−1^ (C–H bending mode in nucleic acids) peak in CKD patients indicates a lower concentration of nucleic acids in their sera. The SERS band of tryptophan at 1450 cm^−1^ showed a lower signal in the CKD group, implying a decrease in phenylalanine levels in these patients. The 890 cm^−1^ peak is assigned to glutathione and D-(C)-galactosamine in the CKD group, but their peaks at 1094, 1278, and 1400 cm^−1^ vanish. The differences or changes between the CKD and normal groups are linked to renal pathological changes and metabolism. The precise mechanisms underlying these spectral changes merit further investigation. With the creation of a training set database and a classification model, PCA-LDA was used to classify healthy subjects and CKD patients. The classification model was further validated using the spectra of independent serum samples from 10 normal subjects and 11 CKD patients, with a sensitivity and specificity of 100% [57]. In a small study of 47 hemodialysis patients and 55 healthy subjects, discrimination of the Raman spectra between normal and dialysis groups, based on PCA with PC2 and PC3, had a sensitivity of 91%, specificity of 98%, and accuracy of 95%. The Raman technique could determine serum urea and serum concentrations with low error and distinguishing hemodialysis from normal subjects [50]. A Chinese case–control study with 126 CKD patients (CKD stages 2–5) and 97 healthy individuals investigated the use of SERS with silver nanoparticles, and multivariate analysis was able to directly diagnose CKD in serum. Both healthy controls and CKD patients had primary SERS peaks at 641, 724, 813, 1003, 1132, 1210, 1326, 1450, 1583, and 1655 cm^−1^. These signals could be attributed to known biochemical components such as L-tyrosine and lactose with C–S vibration structure (641 and 813 cm^−1^), nucleic acids (641, 724, 813, 1003, 1210, and 1450 cm^−1^), carbohydrates (641, 890, and 1094 cm^−1^), lipids (1278 and 1328 cm^−1^), and amino acids (1655 cm^−1^). PCA-LDA discriminated CKD from non-CKD controls with a sensitivity of 74.6%, specificity of 93.8%, and accuracy of 83.0% for the serum spectra. The integration area under the ROC curve was 0.937 ± 0.015 (*p* < 0.0001). The serum spectra separated the various stages of CKD with an accuracy of 78.0% and 75.4%. The serum urea PLS prediction was 0.8540 (*p* < 0.001), the serum creatinine PLS prediction was 0.8536 (*p* < 0.001), and the eGFR prediction was 0.7500 (*p* < 0.001). As a result, SERS could be regarded as a novel tool for detecting CKD in a healthy population. Identification of CKD stage-dependent signals or chemical composition with unknown special nanoparticles is required in future studies for reproducible discrimination between CKD stages [37].

Uremic toxins accumulate in the blood of CKD patients, and their levels are a predictor of cardiovascular events and mortality [58]. Protein-bound uremic toxins (PBUTs), which primarily bind with human serum albumin (HSA) and are not removed by conventional hemodialysis, are a major concern [59,60]. The determination of these compounds during a dialysis session would provide a better understanding of CKD pathology, as well as a diagnostic strategy for predicting disease progression and complications. Monitoring the serum PBUT concentration is also important for comparing the efficacy of therapeutic strategies that have been reported to reduce their plasma concentration in CKD patients’ bloodstreams [61]. PBUTs have been detected using a variety of chromatography-based analytical methods. However, there is a need to create new, simple techniques that can overcome the disadvantages of high-performance liquid chromatography (HPLC) and HPLC-coupled analyses. SERS coupled with an Au nanoparticle substrate has been applied for the simple quantification of 3-carboxy-4-methyl-5-propyl-2-furanpropionate (CMPF) and indole-3-acetic acid (IAA) in human serum samples, which are critical PBUTs. The CMPF and IAA analysis detection limits were estimated to be 0.04 nM and 0.05 µM, respectively. The sensor’s intra-assay precision was evaluated by analyzing IAA at a concentration of 0.020 mg/mL three times, which yielded a similar response and a coefficient of variation of 1.7%. The SERS spectra of CMPF in serum and urine samples from healthy subjects showed the following peaks: (1) serum: 1440 cm^−1^ (CH_3_ and CH_2_ deformation), 1380 cm^−1^ (CH_3_ umbrella mode), 1260 cm^−1^ (stretching vibrations of the C–O bond), 1020 cm^−1^ (C–C), 1000 cm^−1^ (symmetric furan ring torsion); (2) urine: 1458 cm^−1^ (CH_3_ and CH_2_ deformation), 1380 cm^−1^ (CH_3_ umbrella mode), 1260 cm^−1^ (stretching vibrations of the C–O bond), 1000–1050 cm^−1^ (symmetric furan ring torsion), and 1600 cm^−1^ (C=C in the furan ring). Raman bands of IAA in serum and urine from healthy subjects were as follows: (1) serum: 1603 cm^−1^ (vC=C), 1594 cm^−1^ (C–C pyrrole stretch), 1458 cm^−1^ (γC=C in plane), 1434 cm^−1^ (NCC stretch NH bend), 1363–1342 cm^−1^ (Fermi doublet), 1225 cm^−1^ (CH bend NH bend), 1026 cm^−1^ (benzene ring breathing), 996 cm^−1^ (γOH), and 846 cm^−1^ (NH bend); (2) urine: 1575 cm^−1^ (vC=C), 800 cm^−1^ and 1554 cm-1 (C–C pyrrole stretch), 1454 cm-1 (γC=C in plane), 1431 cm^−1^ (NCC stretch NH bend), 1360 cm^−1^ (Fermi doublet), 1010 cm^−1^ (benzene ring breathing), and 995 cm^−1^ (γOH). Individual detection of CMPF and IAA molecules in serum samples revealed nonoverlapping Raman bands with minimal interference from the biological sample matrix. It should be noted that CMPF has prominent SERS peaks near 1380 cm^−1^, 1340 cm^−1^, and 1260 cm^−1^, whereas IAA has prominent SERS peaks near 1026 cm^−1^, 1594 cm^−1^, 1434 cm^−1^, and 1220–1240 cm^−1^. In CKD patients, SERS peaks consistent with serum CMPF and IAA were found at 1325–1375 cm^−1^ (vC–CH_3_ vibrations), 1150 and 1275 cm^−1^ (C–O bonds), and 1510 cm^−1^ (C=C bond). However, because this is a more complicated matrix, some identical SERS peaks may be due to the presence of other non-PBUT uremic toxins, such as creatinine (680 cm^−1^), uric acid (637 and 1138 cm^−1^), and urea (1001 and 1045 cm^−1^). The current method is superior in that it eliminates time-consuming sample treatment steps (analysis time less than 5 min) and reduces the sample limit. Furthermore, SERS has a high sensitivity due to signal enhancers and does not necessitate the use of any specialized scientific instruments [61].

## 4. The Potential Use of Raman Spectroscopy in Specific Kidney Diseases

### 4.1. Anti-Neutrophil Cytoplasmic Autoantibody-Associated Glomerulonephritis

Anti-neutrophil cytoplasmic autoantibody (ANCA)-associated vasculitis is a multisystem autoimmune disease that is caused by necrotizing inflammation of small and medium-sized blood vessels. Renal involvement with ANCA-associated glomerulonephritis is often associated with rapidly progressive disease and a higher mortality risk when compared to patients without renal disease, particularly among those on dialysis [62]. Renal biopsy is still the gold standard for diagnosing ANCA-associated glomerulonephritis, but its serial use for disease monitoring is limited due to procedural risks and resource requirements. Interobserver variability in key histological findings such as interstitial fibrosis and tubular atrophy (IFTA) and interstitial infiltrate is possible, with the former having important prognostic implications. As a result, there is still room for adjuvant techniques to supplement and aid current issue analysis [63]. In a pilot study of 11 patients with a new diagnosis of ANCA-associated glomerulonephritis and 17 in disease remission, the role of RS as a method for automated computational detection of disease activity was evaluated. After supervised classification based on recorded histological data, spectral data from unstained tissue samples were able to discriminate disease activity with a high degree of accuracy on blind predictive modeling: F-score 95% for >25% interstitial fibrosis and tubular atrophy (sensitivity 100%, specificity 90%), 100% for necrotizing glomerular lesions (sensitivity 100%, specificity 100%), and 100% for interstitial infiltrate (sensitivity 100%, specificity 100%). According to these findings, spectral data correctly differentiated the presence of histological lesions indicative of chronic damage and active disease, such as IFTA, interstitial infiltrate, and necrotizing glomerulonephritis. The wavenumber variables that were responsible for the greatest between-group differences in the first two were associated with increased amino acid and cortisol activity, whereas IFTA was associated with increased nucleic acid expression. Thus, biospectroscopy provides a potentially novel machine learning method for automated computational detection of ANCA-associated glomerulonephritis disease activity in renal biopsy specimens. A subgroup analysis was also performed to see if urine spectral data could be used as a surrogate for renal biopsy, with the premise being that the biomolecular signature obtained from urine could characterize and reflect histological findings at a given timepoint. Using the same chemometric methodology, the model’s ability to reliably distinguish the presence of necrotizing glomerulonephritis lesions, interstitial infiltrate, and IFTA in urine was limited, with poor sensitivity in each group. This could be due to the subgroup’s small sample size in each category and the possibility of insufficient training data. Taking this into consideration, further research in this area should not be discouraged. Any future study evaluating the role of biospectroscopy in ANCA-associated glomerulonephritis would benefit from assaying and analyzing classical (C-reactive protein (CRP), anti-myeloperoxidase (MPO, p-ANCA)/anti-proteinase-3 titers (PR3, c-ANCA) antibodies) and potential biomarkers (including urinary monocyte chemoattractant protein-1 (uMCP-1), urinary soluble CD163 (sCD163), and complement cascade degradation products) with spectral data [64,65,66,67,68,69,70,71]. Other potential research areas include the use of forward feature extraction algorithms to build prediction outcome models based on extracted spectral features, as well as the correlation of spectral data with imaging mass spectrometry to aid in the identification of potential biomarkers [1]. 

### 4.2. Primary Focal Segmental Glomerulosclerosis

Focal segmental glomerulosclerosis (FSGS) is a morphologic pattern of glomerular injury defined by sclerosis in parts (segmental) of some (focal) glomeruli and global podocyte foot process effacement in all glomeruli. There are three types of FSGS: primary (idiopathic), secondary, and genetic. Despite sharing clinical and histologic characteristics, these subclasses differ in terms of management and prognosis [72]. Although primary FSGS is a rare disease, it is one of the most common nephropathies causing ESKD. Circulating permeability factors are most likely to blame. Despite decades of research and several identified potential factors, no unifying concept for circulating factors has been established [73]. There is an unmet clinical need for early and specific biomarkers to detect FSGS and predict prognosis and treatment response. Until now, diagnostic and therapeutic decisions have been based on nonspecific markers such as proteinuria, serum creatinine, and renal histology. However, because FSGS is a focal disease prone to biopsy sampling errors, the actual disease of the patients may be misinterpreted even by histology [74]. As a result, there is a greater demand in FSGS research for the use of novel technologies that enable us to study FSGS from previously unexplored angles. Raman microspectroscopy might be able to provide a molecular fingerprint of FSGS at the tissue level. This hypothesis was tested in an FSGS patient who received a living-related kidney transplant and had recurrent FSGS in the transplanted kidney. Although all of the classically published circulating factors were within normal limits, an immediate response to CytoSorb apheresis was suggestive of pathogenic circulating factors. A podocyte cell culture model and a proteinuria model in zebrafish were used to prove the functional effects of the patient’s serum on podocytes and the glomerular filtration barrier. RS was performed on podocytes treated with the patient’s serum and healthy control serum, <50 kDa serum fractions of the FSGS patient, and four healthy control patients, in addition to renal biopsies of a patient at the time of kidney transplantation, a patient with FSGS recurrence, and a patient with minimal change disease. At the time of disease recurrence, FSGS patient samples contained Raman signals corresponding to changes in lipid concentrations, as well as lipid composition. The most noticeable differences in Raman peaks between FSGS serum- and control serum-treated cultured human podocytes were found at wavelengths corresponding to membrane-bound phosphatidylcholine, phenylalanine, phospholipids, fatty acids, and sphingomyelin. The Raman spectra of the <50 kDa serum fraction of the FSGS patient at the time of recurrence corresponded to phospholipids, phosphatidylcholine, and sphingomyelin, confirming a lipoprotein profile dysbalance. The FSGS kidney biopsy’s Raman signal also revealed increased membrane-bound phosphatidylcholine, phenylalanine, phospholipids, fatty acids, and sphingomyelin, which were consistent with FSGS’s disrupted systemic and renal lipid expression. Dyslipidemia is a common finding in nephrotic syndrome, which includes FSGS. Several serum metabolomic signatures involved in lipid metabolism disruptions in FSGS have been identified, corresponding to FSGS serum Raman signals, serum-treated podocyte Raman signals, and biopsy after FSGS recurrence Raman signals. These lipoprotein changes may reveal new pathways involved in the pathomechanism of recurrent FSGS [75]. Major Raman peaks for albumin (830 cm^−1^, 950 cm^−1^, 1350 cm^−1^, and 1650 cm^−1^ [76]) were higher in the FSGS relapse biopsy than in the baseline biopsy, indicating a higher albumin abundance in the damaged kidney due to glomerular filtration barrier leakage [75]. Furthermore, increased collagen along the Bowman’s capsule has been reported in FSGS mice [77], and the Raman measurements in the FSGS relapse biopsy showed an increased signal at 1259 cm^−1^ [75]. A machine learning-based anomaly detection method was also used to identify differences in the Raman spectra between the baseline biopsy and the FSGS recurrence biopsy. In the FSGS recurrence kidney biopsy, differences were found in the area of parietal epithelial cells and the focal points of the glomerulus. Cuboidal parietal cells, a novel parietal epithelial cell subpopulation colocalized with the Bowman’s capsule, were recently proposed to form tip lesions in FSGS [78]. In line with this, parietal epithelial cell activation has been described in early recurrent FSGS [79]. Focal activation of parietal epithelial cells contributed to the development and progression of sclerotic lesions in three different FSGS models and human biopsies with FSGS [80]. 

### 4.3. Anti-Glomerular Basement Membrane Disease

Anti-glomerular basement membrane (GBM) disease, also known as Goodpasture’s disease, is a relatively uncommon cause of glomerulonephritis caused by autoantibodies directed at specific antigenic targets within the glomerular or pulmonary basement membrane. In the mouse model of this disease, acute exposure to “nephrotoxic” anti-GBM antibodies can rapidly and reproducibly impair the recipient mice’s renal function. The efficacy of using RS combined with multivariate analysis as a diagnostic tool for discriminating healthy kidneys from kidneys afflicted with acute nephritis from anti-GBM mouse models has been explored. Dominant Raman bands were observed among normal and diseased tissue corresponding to putative biochemicals such as phenylalanine at 1000 cm^−1^, collagen at 1265 cm^−1^, lipid at 1442 cm^−1^, and amide I at 1647 cm^−1^. By applying multivariate analysis to RS, researchers were able to distinguish between the diseased and the non-diseased mice with up to 100% accuracy, and between the severely diseased [129/svJ (129)], mildly diseased [C57BL/6 (B6)], and healthy mice with up to 98% accuracy. It has the potential to largely reduce the complexity of diagnosing and monitoring anti-GBM disease. This model could also be used to investigate the downstream pathways that lead to chronic nephropathies such as lupus nephritis [2]. 

### 4.4. Diabetic Kidney Disease

Diabetic kidney disease (DKD) is one of the most common complications of type 2 diabetes and the leading cause of ESKD worldwide [81]. It is a microvascular complication characterized by an increase in urine albumin excretion (UAE), glomerular lesions, and a decrease in eGFR [82]. The UAE remains the cornerstone for DKD diagnosis and classification [83]. As the diabetes epidemic spreads, particularly in low-income countries [84], there is a growing demand for low-cost alternatives to immunochemical assays, nephelometry, immunoturbidimetry, enzyme-linked immunosorbent assays, radioimmunoassay, and high-performance liquid chromatography (HPLC) to measure the UAE [85]. 

Recently, RS was used to detect albumin in artificial and human urine samples. An increased intensity at the peak of 1450 cm^−1^ and 1630 cm^−1^ was detected in artificial samples with higher urinary albumin concentrations using prolonged exposure and fluorescence background removal using a wavelets-based method [33]. An increased intensity at the peak of 1450 cm^−1^ may correspond to amino-acid glycine [86] concentration in albumin, representing a Raman window for the detection of the presence and concentration of albumin in the urine when analyzing the Raman spectra obtained from artificial urine by increasing the concentration of albumin. When albumin was dissolved in sterile water, one peak overlapped with the Raman signal of water at 1630 cm^−1^, which also represents the highest albumin concentration. Nonetheless, the mix of water with albumin presented another detection peak at 1431 cm^−1^, which reinforces the idea that RS can be a useful tool to detect albumin in water dissolutions. In urine samples from type 2 diabetes mellitus patients, the presence of albumin was found at the peaks of the spectrum at 663, 993, 1021, 1235, 1430, and 1634 cm^−1^ [33]. 

In a model of obese diabetic ZSF1 rats with kidney disease fed with whole grape powder containing chow or control chow, PCA/LDA of the SERS spectra of urine samples was able to separate the grape-fed group from controls with 72.7% sensitivity and 60% specificity. The integration area under the curve was 0.800 ± 0.097 (*p* = 0.02), according to the ROC curves [44]. The major differences between the whole grape powder chow and control chow groups were found at 662, 733, 912, 1000, 1323, and 1674 cm^−1^, with 1000 cm^−1^ being the most significant [7]. Direct SERS has the sensitivity required to detect clinically relevant urinary albumin concentrations, a strategy that could be used in the future for point-of-care microalbuminuria screening. In a proof-of-concept study, 27 urine samples (with urinary albumin concentrations from 0 to 120 μg/mL) were analyzed by SERS with iodide-modified silver nanoparticles. Using PCA-LDA and cutoff values of 3, 6, and 10 g/mL, groups with high and low urinary albumin concentrations could be discriminated with an overall accuracy of 89%, 93%, and 89%, respectively. On the basis of the 1002 cm^−1^ SERS band, which is attributed to the ring breathing vibration of phenylalanine, a detection limit of 3 g/mL for human serum albumin was reported. This detection limit is comparable to immunoturbidimetric assays and is approximately one order of magnitude lower than urinary detection limits. The R^2^ and RMSE of prediction between predicted and reference values of human serum albumin concentrations were 0.982 and 2.82, respectively, using an independent prediction set. As a result, there was an excellent correlation between predicted and reference values, highlighting SERS’s potential for absolute albumin quantification. Furthermore, because of its high sensitivity and specificity, this method could be used to assess small changes in UAE concentrations, resulting in more efficient treatment monitoring to slow or even stop the progression to macroalbuminuria. When compared to immunoturbidimetric analysis or HPLC, SERS spectroscopy is faster. However, translating this method into a clinical setting will necessitate several additional steps that address variations in urine chemical composition, as well as a prospective validation in large clinical trials [39].

Extracellular vesicles are a diverse population of bilayer cell membrane structures with sizes ranging from 30 to 1000 nm that are released into the extracellular space. They transport a variety of biomolecules, including proteins, nucleic acids, lipids, and metabolites, which can be transferred to recipient cells [87,88]. Urinary extracellular vesicles’ molecular content may reflect the physiological and pathological state of the epithelial cells that form nephrons and the lower urinary tract [89,90]. RS could detect subtle differences between the spectral fingerprints of urinary extracellular vesicles derived from urine samples of healthy subjects and patients with diabetes mellitus diagnosed with different stages of CKD using multivariate statistical methods such as PCA and partial least squares regression (PLSR). The bands originating from tryptophan T (746–775 cm^−1^), phenylalanine P (995–1011 cm^−1^), and amide I (1629–1709 cm^−1^), which were positive peaks in the PC1 loading plot, made the greatest contributions to the discrimination of the studied groups. The bands derived from proteins and lipids were negative peaks in the PC1 loading plot: C–N stretching (1115–1143 cm^−1^), C–H bending (1404–1503 cm^−1^), and lipids (1282–1305 cm^−1^) [91]. Following the calculation of variable importance in projection values using the PLS model, the following ranges were considered for further analysis: protein bands (746–775 cm^−1^ and 995–1011 cm^−1^), lipid bands (1055–1070 cm^−1^, 1282–1305 cm^−1^, 1429–1448 cm^−1^, and 1710–1774 cm^−1^), and protein and lipid bands (1115–1143 cm^−1^ and 1661–1699 cm^−1^) [92,93,94,95,96]. The highest correlations were found between diabetes mellitus duration and eGFR, as well as the area under characteristic Raman bands for tryptophan (747–775 cm^−1^; R = 0.78, R = 0.76) and amide III (1190–1270 cm^−1^; R = 0.78, R = 0.74). In a regression model with eGFR as the dependent variable, tryptophan and amide III as independent variables had the highest adjusted R^2^ values, explaining 78% of the variation. These findings show that the protein and lipid components of urinary extracellular vesicles change with CKD stage. In the future, Raman spectral signatures of urinary extracellular vesicles could represent a reliable and precise method for stratifying CKD patients and possibly evaluating the efficacy of pharmacological treatments [91].

### 4.5. Kidney Transplantation

Kidney transplantation, which provides better survival and quality of life than dialysis, has been the preferred treatment for patients with ESKD. Due to the global challenge of donor kidney shortage, it has been proposed to expand the pool of deceased donors to include expanded criteria donors. However, the lack of methods for precisely measuring donor kidney injury and predicting outcome continues to result in high discard rates and recipient complications. As a result, assessing the quality of deceased donor kidneys before transplantation is critical. Clinical scores, kidney biopsy histopathology, and machine perfusion systems are the most commonly used methods [97]. However, clinical scoring systems are generally inaccurate, resulting in unnecessary organ discard. Because of the relatively weak association with kidney quality, machine perfusion parameters are not suitable as standalone criteria, and kidney biopsies are invasive [98]. Biomarkers [e.g., neutrophil gelatinase-associated lipocalin (NGAL), kidney injury molecule-1 (KIM-1), liver-type fatty acid binding protein (L-FABP), monocyte chemoattractant protein-1 (MCP-1), chitinase-3-like 1 (YKL-40), interleukin 18 (IL-18), secretory leukocyte peptidase inhibitor (SLPI), etc.] derived from donor urine or serum may offer advantages in the precise measurement of kidney quality [99,100,101,102]. RS and SERS have been evaluated as alternative tools because there is currently no gold-standard method for detecting biomarkers involved in donor kidney injury. Using BP/Au nanohybrids as Raman enhancing generators, a novel label-free and highly sensitive SERS detection strategy for biomarkers related to the quality of a kidney transplant has been proposed. SERS band intensities at 950 cm^−1^ [C–H bending/v(C–H)/v(C–CH_3_)] and at 1000 cm^−1^ (Phe v12 and ρCH2) [103,104,105] were linked to IL-18 and SLP, respectively. The detection limits for SLPI were 1.53 × 10^−8^ mg/mL and 0.23 × 10^−8^ mg/mL for IL-18. The quantification limits for SLPI and IL-18 were 5.10 × 10^−8^ mg/mL and 7.67 × 10^−9^ mg/mL, respectively. Given that the combined assessment of SLPI and IL-18 expression levels serves as an indicator of donor kidney quality and can be performed quickly, quite simply, and reproducibly, this SERS-based method has great clinical potential [98].

Urine SERS measurements from deceased donors and associated PCA-LDA analysis could predict kidney transplant outcomes. PCA-LDA and logistic regression achieved more than 90% sensitivity in differentiating high donor acute kidney injury (AKI) risk with acceptable transplant outcomes [acute tubular necrosis (ATN)] from those with recipient delayed graft function (DGF). Both the ATN and the DGF classes had distinct spectral features that allowed them to be distinguished from the control class. The presence of recipient DGF resulted in the most intense spectral differences from the control class, whereas the presence of donor ATN affected more subtle features of SERS spectra. SERS has the potential to provide an early indication of deceased donor kidney transplant outcome before transplantation and can be a valuable tool for clinicians in expanding the deceased donor kidney pool by utilizing kidneys with high donor AKI risk as detected by other biomarkers [106].

In a study with 110 kidney transplant recipients, PLS analysis of the urine’s silver nanoparticle-based SERS spectra revealed a significant relationship among urine protein (R^2^ = 0.4660, *p* < 0.01), creatinine (R^2^ = 0.8106, *p* < 0.01), and urea (R^2^ = 0.7808, *p* < 0.01). In the presence of uncertainty (remaining kidney function and therapeutic drugs) and other unknown factors, the urine SERS spectra were capable of predicting essential kidney biomarkers. The finding that silver nanoparticle-based SERS spectra of the urine can predict kidney transplant function should be further validated in larger cohorts [11].

Although the occurrence of acute rejection (AR) following kidney transplant has decreased over the last decades, AR remains a major cause of renal transplant failure. Serum creatinine is the most common, but not the ideal biomarker for assessing kidney function. As a kidney biopsy is still the gold standard for the diagnosis of an AR, there is a call for the development of early, rapid, cheap, sensitive, and specific biomarkers for the detection of graft failure. The utility of SERS-based urine measurements for early detection of potential AR in kidney transplant patients has been studied. In small pilot studies [45,107], the urine spectra of all AR patients had a very intense peak at 1360 cm^−1^ and two peripheral peaks at 1448 and 1495 cm^−1^, whereas the 1360 cm^−1^ peak was either absent or very weak in most normal patients’ spectra. The pyrrole half-ring symmetrical stretch within the free heme molecule is responsible for the intense peak at 1356–1376 cm^−1^. SERS analysis is more specific to abnormal kidney function than serum creatinine, which may reduce the use of biopsies, resulting in less patient discomfort and lower medical costs. 

The activated T lymphocytes are the predominant immune cells involved in the process of AR. In an in vitro study of an acute allograft rejection model, RS could distinguish between activated and nonactivated T lymphocytes and, thus, establish distinct signatures for both. A preliminary qualitative comparison of activated and nonactivated T lymphocytes revealed differences at four distinct points in the Raman spectrum: 788, 1000, 1182, and 1195 cm^−1^ [108]. Peaks at 788 and 1000 cm^−1^ have previously been identified and described as indicators of cellular viability [109]. Peak positions 1182 and 1195 cm^−1^ (corresponding with tyrosine/phenylalanine and adenine/thymine changes) had a very distinct spectral difference [108,110]. RS was further used to examine 75 inactivated, 40 alloantigen-activated, and 75 CD3/CD28-activated T cells. CD3/CD28 activated peak magnitudes (PM) were 4.3–23.9% lower than inactivated PM at the following positions: 903, 1031, 1069, 1093, 1155, 1326, and 1449 cm^−1^, with a difference in peak ratio (PR) observed at the 1182:1195 position (*p* = 0.006). Although the primary cause of this pattern is unknown, it is most likely a reshuffling rather than a downregulation of cell surface bio-molecular material. There were differences in CD3/CD28- and alloantigen-activated PM at 903, 1031, 1093, 1155, 1326, and 1449 cm^−1^. This Raman shift is most likely a conserved change in cell surface molecules shared by activated T cells regardless of activation method. When the 1182:1195 peak ratios were examined, similar patterns were discovered in both the alloreactive and the CD3/CD28-activated samples that were not present in the inactivated or resting T-cell samples. The separation of CD3/CD28- and alloantigen-activated groups by spectral signature was 100% specific and sensitive. Changes in nucleic acids are represented by 903, 1093, and 1449 cm^−1^, and changes in amino acids are represented by 1031 and 1155 cm^−1^, whereas both nucleic acid and amino acid changes are represented by 1326 cm^−1^, consistent with molecular processes responsible for the transcription, translation, and expression of cell surface receptors [110].

The use of hydroxyethyl starch (HES) in brain-dead donor resuscitation has been linked to the presence of osmotic-nephrosis-like lesions in kidney transplant recipients [111]. However, the presence of HES in the kidney could also be detected in the absence of osmotic nephrosis-like lesions, indicating that the accumulation of HES in the kidney does not always affect the histological aspect of tubular cells as can be seen using light microscopy. RS has been used for detecting HES in kidney biopsies from patients who received HES during a collapse and developed AKI with osmotic nephrosis lesions [112]. HES accumulation in donor’s kidneys, as measured by Raman microscopy, has been related to donor kidney quality and, thus, to graft renal function (r = 0.88; *p* < 0.001) and survival (r = −0.72; *p* < 0.001). In protocol renal graft biopsies 3 months after transplantation (HES group = 20, control group = 6), HES compounds could be detected using this label-free technique in 40% of the HES group. The association between high HES signaling in renal tubular cells and better renal graft quality may appear to contradict the molecule’s renal toxicity. However, although an increasing HES signal was associated with a lower risk of graft failure, this was primarily due to fewer donor morbidities and, as a result, better renal graft quality [111].

## 5. Conclusions

RS allows the simultaneous analysis of a wide range of biomolecules, serving as an adjuvant technique for biomarker extraction and providing additional potential insight into the disease’s molecular mechanisms [1]. This method can give a molecular fingerprint of kidney diseases and reveals metabolomic changes corresponding to serum mass spectrometry findings [75]. The combination of RS and other optical methods within a single portable device expands the analysis and, in theory, allows for the identification of various pathological conditions of the human body. To achieve this goal, however, it will be necessary to conduct experimental work with a larger sample set of patients with various pathological conditions, as well as to expand the sample set of healthy people [18]. The RS data should be compared to the gold standards currently used in nephrology. For example, when using RS to estimate GFR, the bias and accuracy (P30) should be detailed in comparison to a gold-standard measure of GFR (inulin clearance, iothalamate, or iohexol). 

## Figures and Tables

**Table 1:** Raman spectroscopy (RS) and surface-enhanced Raman spectroscopy (SERS) studies for measuring creatinine, urea, glucose, protein/albumin, and blood in urine.

Parameter	Method	Findings	References
Creatinine	RS	Peak at 692 cm^−1^ R = 1.00 LOD = 1.5 mg/mL	[29]
	Gold colloid SERS	Peak between 1390 and 1490 cm^−1^ R^2^ = 1.0	[38]
	RS	R^2^ > 0.98 RMSEcv = 4.9 mg/dL	[30]
	RS and a Teflon^®^ liquid core optical fiber-based excitation–collection geometry	Peak at 687.5 cm^−1^ RMSEcv = 6.2 mg/dL	[27,28]
	Silver nanoparticle-based SERS	Peak at 1400–1500 cm^−1^ R^2^ = 0.96	[35]
	SERS with metalized nanostructure parylene (PPX-Cl) film as a SERS substrate	Peaks at 700, 840, 900, and 1420 cm^−1^ 840 and 900 cm^−1^ peaks were stronger than the 700 cm^−1^ peak R = 0.907 for 840 cm^−1^, 0.968 for 900 cm^−1^	[36]
	RS	Peak at 608, 680, 846, and 910 cm^−1^	[32]
	RS	Peak at 527, 1006, and 1160 cm^−1^ R = 0.91 RMSEcv = 25.2 mg/dL	[31]
	AuNP coated Blu-ray DVD (BRDVD)-based SERS	Peak at 888, 958, and 1444 cm^−1^ LOD: 0.2 µg/mL	[34]
	RS	Peak at 650–940 cm^−1^	[33]
	Silver nanoparticle-based SERS	R^2^ = 0.81	[11]
	Silver nanoparticle-based SERS	R^2^ = 0.79 RMSEcv = 4.19 mmol/L	[37]
Urea	RS	Peak at 1003 cm^−1^	[26]
	RS and a Teflon^®^ liquid core optical fiber-based excitation-collection geometry	Peak at 1014 cm^−1^ RMSEcv = 39.5 mg/dL	[27,28]
	RS	Peak at 1016 cm^−1^ R = 0.97 LOD: 20 mg/dL	[29]
	RS	Peak at 1000 cm^−1^	[30]
	RS	Peak at 681, 846, and 908 cm^−1^ R = 0.90 RMSEcv = 312 mg/dL	[31]
	RS	Peak at 1004 cm^−1^	[32]
	RS	Peak from 500–560 cm^−1^, from 960–1043 cm^−1^, from 1120–1192 cm^−1^	[33]
	AuNP coated Blu-ray DVD (BRDVD)-based SERS	Peak at 1018 cm^−1^ LOD: 0.6 µg/mL	[34]
	SERS with metalized nanostructure parylene (PPX-Cl) film as a SERS substrate	Peak at 1000 cm^−1^	[36]
	Silver nanoparticle-based SERS	Peak at 1004 cm^−1^ R^2^ = 0.78	[11]
	Silver nanoparticle-based SERS	R^2^ = 0.73 RMSEcv = 632.44 mmol/L	[37]
	Gold colloid SERS	Peak at 1000 cm^−1^	[38]
Glucose	RS	Peak at 1130 cm^−1^ R = 1.00 LOD: 32 mg/dL	[29]
	RS	Peak at 1128 cm^−1^	[32]
Protein/albumin	RS	Peak at 600, 850, 1060, and 1470 cm^−1^ (protein) R^2^ = 0.97	[26]
	Silver nanoparticle-based SERS	Peak at 1002 cm^−1^ (albumin) R^2^ = 0.98 LOD of albumin: 3 µg/mL	[39]
	SERS coupled with GONR catalysis	Peak at 1615 cm^−1^ (albumin) LOD of albumin = 0.02 ng/mL	[40]
	AuNP-coated Blu-ray DVD (BRDVD)-based SERS	Peak at 1208 and 1370 cm^−1^ (albumin) LOD of albumin: 0.1 µg/mL	[34]
	AGMS device coupled with SER	R^2^ = 0.99 LOD of albumin = 2.0 mg/L	[41]
	Polydopamine bifunctionalized glass chip and SERS	Peak at 1096 cm^−1^ R^2^ = 0.99 LOD of albumin: 0.2 mg/L	[42]
	RS	Peak at 1450 cm^−1^ (albumin)	[33]
	Silver nanoparticle-based SERS	Peak at 890 cm^−1^ (protein) R^2^ = 0.47	[11]
	Silver nanoparticle-based SERS	R^2^ = 0.6579 (albumin) RMSEcv = 10.50 mmol/L	[37]
Blood	RS	R^2^ = 0.91 (all hematuria levels), 0.92 (microhematuria)	[43]

Abbreviations: RS, Raman spectroscopy; R, correlation coefficient; ; LOD, limit of detection; SERS, surface-enhanced Raman spectroscopy; R^2^, coefficient of determination; RMSEcv, root-mean-squared error of cross-validation; GONR, graphene oxide nanoribbon; AuNP, gold nanoparticle; AGMS, array gas membrane separation.

**Table 2:** Research into the use of Raman spectroscopy (RS) and surface-enhanced Raman spectroscopy (SERS) to measure serum creatinine and urea.

Parameter	Method	Finding	References
Creatinine Creatinine	Silver nanoparticle-based SERS	Peak between 530 and 1070 cm^−1^ LOD < 0.1 µg/mL RMSEP = 0.0065 (1.3%, 6 PLS factors), 0.014 (0.3%, 8 PLS factors)	[52]
	RS	Peak at 680 and 846 cm^−1^ R = 0.93 RMSEcv = 1.94 mg/dL	[50]
	Nano-Au on Ag film SERS	Peak at 678 cm^−1^ LOD = 4.42 × 10^−3^ µmol/mL	[55]
	Silver nanoparticle-based SERS	R^2^ = 0.76	[11]
	Silver nanoparticle-based SERS	R^2^ = 0.85 RMSEcv = 31.83 mmol/L	[37]
	Silver nanoparticle-based SERS chip integrated with a MOS	Peak at 678 cm^−1^ R^2^ = 0.91	[53]
	Nano-Ag/Au@Au film composite SERS	Peak at 612 cm^−1^ R = 0.96 LOD = 5 × 10^−6^ mol/L	[54]
	RS	Peak at 681 and 846 cm^−1^ R = 0.64 RMSEcv = 0.21 mg/dL (PLS-based regression model)	[51]
	Au nanoparticle-based SERS	Peak at 685 cm^−1^ R = 0.99	[56]
Urea	LCOF RS	Peak from 510–1800 cm^−1^ RMSEcv = 2.2 mg/dL	[28]
	RS	Peak at 1004 cm^−1^ R = 0.97 RMSEcv = 17.6 mg/dL	[50]
	Silver nanoparticle-based SERS	R^2^ = 0.65	[11]
	Silver nanoparticle-based SERS	R^2^ = 0.85 RMSEcv = 2.47 mmol/L	[37]
	RS	Peak at 1004 cm^−1^ R = 0.89 RMSEcv = 4.9 mg/dL (PLS-based regression model)	[51]

Abbreviations: SERS, surface-enhanced Raman spectroscopy; LOD, limit of detection; RMSEP, root-mean-square error of prediction; RS, Raman spectroscopy; R, correlation coefficient; RMSEcv, mean square error of cross-validation; Au, gold; Ag, silver; R^2^, coefficient of determination; MOS, micro-optical system; LCOF, liquid-core optical fiber.

## Data Availability

Not applicable.
